# The degree of consistency of applying parental dietary and sedentary behavior rules as indicators for overweight in children: a cross-sectional study

**DOI:** 10.1186/s12889-022-12742-8

**Published:** 2022-02-18

**Authors:** Emilie L. M. Ruiter, Gerdine A. J. Fransen, Marloes Kleinjan, Koos van der Velden, Gerard R. M. Molleman, Rutger C. M. E. Engels

**Affiliations:** 1 Integrated Health Policy, Primary and Community Care, Academic Collaborative Center AMPHI, Radboud University Medical Center, ELG 117, P.O. Box 9101, 6500 HB Nijmegen, the Netherlands; 2 Trimbos Institute, P.O. Box 725, 3500 AS Utrecht, the Netherlands; 3 Interdisciplinary Social Science, University Utrecht, P.O. Box 80.140, 3508 TC Utrecht, the Netherlands; 4 Academic Collaborative Centre AMPHI, Primary and Community Care, Radboud University Medical Centre, ELG 117, P.O. Box 9101, 6500 HB Nijmegen, the Netherlands; 5 Department of Psychology, Erasmus University Rotterdam, P.O. Box 1738, 3000 DR Rotterdam, the Netherlands

**Keywords:** Parental rules, Latent class analyses, Parenting, Overweight prevention, Children, Dietary and sedentary behavior

## Abstract

**Background:**

Review studies increasingly emphasize the importance of the role of parenting in interventions for preventing overweight in children. The aim of this study was to examine typologies regarding how consistently parents apply energy-balance related behavior rules, and the association between these typologies and socio-demographic characteristics, energy balance-related behaviors among school age children, and the prevalence of being overweight.

**Methods:**

For this cross-sectional study, we had access to a database managed by a Municipal Health Service Department in the Netherlands. In total, 4,865 parents with children 4–12 years of age participated in this survey and completed a standardized questionnaire. Parents classified their consistency of applying rules as “strict”, “indulgent”, or “no rules”. Typologies were identified using latent class analyses. We used regression analyses to examine how the typologies differed with respect to the covariates socio-demographic characteristics, children’s energy balance-related behaviors, and weight status.

**Results:**

We identified four stable, distinct parental typologies with respect to applying dietary and sedentary behavior rules. Overall, we found that parents who apply “overall strict EBRB rules” had the highest level of education and that their children practiced healthier behaviors compared to the children of parents in the other three classes. In addition, we found that parents who apply “indulgent dietary rules and no sedentary rules” had the lowest level of education and the highest percentage of non-Caucasians; in addition, their children 8–12 years of age had the highest likelihood of being overweight compared to children of parents with “no dietary rules”.

**Conclusions:**

Parents’ consistency in applying rules regarding dietary and sedentary behaviors was associated with parents’ level of education and ethnic background, as well as with children’s dietary and sedentary behaviors and their likelihood of becoming overweight. Our results may contribute to helping make healthcare professionals aware that children of parents who do not apply sedentary behavior rules are more likely to become overweight, as well as the importance of encouraging parents to apply strict dietary and sedentary behavior rules. These results can serve as a starting point for developing effective strategies to prevent overweight among children.

## Background

Childhood overweight and obesity are a major public health concern worldwide [1, 2], particularly among children in low socio-economic status (SES) families. Moreover, in the Netherlands, being overweight and obese is most prevalent among children of Turkish or Moroccan descent [3–5]. Addressing this increasing problem and preventing overweight are important because overweight and obesity is typically rather difficult to treat [6] and because of their far reaching (social) health consequences that can develop in childhood and/or adulthood [7–9]. A high number of children have unhealthy energy-balance-related behaviors (EBRBs), including low consumption of fruit and/or vegetables, high intake of sugar-sweetened beverages (SSBs), and amounts of excessive screen time. [10–13]. Moreover, over the long term unhealthy EBRBs can result in children becoming overweight or obese. In addition, review studies show that a high-fiber diet, for example a diet containing high amounts of fruits and vegetables consumption, and regular physical activity (PA) have been shown to be protective against becoming overweight and obese [3, 5].

Improvement of the EBRBs can contribute to the prevention of childhood obesity, but requires understanding of the factors determining children’s EBRBs. Parents play an essential role in the development of their child’s EBRBs by facilitating, promoting, and modeling EBRBs in their child's microenvironment, and by dealing with various environmental factors that contribute to the risk of overweight or obesity [14, 15]. Several reviews emphasize the importance of parenting in developing healthy EBRBs and helping prevent childhood overweight and obesity [16–19]. In addition, improving parenting skills (both general parenting styles and specific EBRB practices) can increase the efficacy of interventions designed to prevent overweight or obesity in children [17, 20]. Examples of healthy EBRBs in school age children include the daily consumption of breakfast, eating fruits and vegetables, drinking fewer than two glasses of SSBs a day, engaging in less than two hours of screen time a day, and playing outside for at least one hour a day [21–23].

In addition to the moderating role that general parenting styles play in EBRBs [24–28], establishing specific parental EBRB rules can have a positive effect on the child’s healthy dietary and physical activity behaviors while reducing the child’s television watching and unhealthy dietary behaviors [29, 30]. Furthermore, research show that unhealthy behaviors are more common among children of parents who do not apply EBRB rules. For example, absence of rules regarding watching television is associated with an average of > 2 h of television watching per day [31]. Thus, establishing and applying EBRB rules seems to be important steps for preventing the child from developing a positive energy balance state, which can lead to overweight. Therefore, it is important to involve parents in interventions and to emphasize the importance of applying EBRB rules. In addition, regarding the likelihood of a child becoming overweight, Leech reviewed studies to identify clustering patterns (typologies) of diet, PA and sedentary behavior among children or adolescents and their associations with socio-demographic indicators, and overweight and obesity. He found that obesogenic EBRBs cluster among school-age children in complex ways, that are both conducive and deleterious to good health, and thereby might have a cumulative effect on the development of overweight [32]. Because EBRBs seem to cluster, we hypothesized that this clustering phenomenon might also apply to EBRB rules established by parents. This may have implications for public health because understanding which EBRB rules need to be targeted simultaneously is useful to assist in the development of overweight prevention initiatives.

To increase our understanding of the effect of parental EBRB rules on the likelihood of a school age child becoming overweight, our aim was to examine: *i*) typologies regarding the degree of consistency with respect to applying parental EBRB rules across dietary and sedentary behavioral domains; *ii*) the association between these typologies and socio-demographic characteristics; *iii)* the association between these typologies and healthy or unhealthy EBRBs among children; and *iv*) the association between these typologies and the prevalence of overweight among children. These results may help identify risk profiles of parents who could be targeted for preventive interventions, and the results may help improve existing interventions designed to prevent childhood overweight and obesity.

## Methods

### Study design

This study was a cross-sectional, survey-based study.

### Database

For our study, we had access to the Child Monitor, a database managed by the Municipal Health Services Department of the Gelderland-South region in the Netherlands. This database we also used in another published study. For that reason we will describe this method section in an uniform way to that study [33]. This database contains data collected in a cross-sectional survey conducted in 2009, to gain insight into the population’s health, lifestyle and well-being of children age 6 months to 12 years living in the Nijmegen region of the Netherlands [33, 34]. The Child Monitor is part of a monitoring cycle and is required by the Dutch Public Health Act. For the purpose of our study, we used data collected from parents with a child 4–12 years of age at the time of the survey, as the questions in the Child Monitor questionnaire about establishing EBRBs rules were only for parents of children 4–12 years of age. The Child Monitor questionnaire is part of the "Local and National Health Monitor", which uses standard questions. The questionnaire was available only in Dutch. Parents who have difficulty with the Dutch language were able to request help with completing the questionnaire.

Because these data were routinely collected and are anonymous, and because participation in the survey was voluntary, informed consent was not required for this study [33]. The research population that was asked to complete the Child Monitor questionnaire was well informed about the aim of the Child Monitor [33]. The design and method (data collection) of the Child Monitor complies with the legal provisions of the Personal Data Protection Act and the General Data Protection Regulation in the Netherlands and the protocol was approved by the institutional review board of the Municipal Health Services, Department of the Gelderland-South region in the Netherlands [33]. We received approval from the Director of the medical office of the Municipal Health Services Department of the Gelderland-South region in the Netherlands to use the Child Monitor database for our analyses and to report the results [33].

### Participants

In 2009, a total of 15,991 parents/caregivers in the Gelderland-South region of the Netherlands with a child between the ages of 6 months and 12 years were randomly selected and invited to complete the Child Monitor questionnaire (either digitally or on paper). A total of 9,796 parents (61%) completed the questionnaire, and 4,865 of these questionnaires were about children 4–12 years of age at the time the questionnaire was completed.

### Measures

#### Socio-demographic characteristics

The following demographic data were obtained from parents via the Child Monitor questionnaire: the child’s age, gender, and ethnicity, and the level of education of both parents (as an indicator of the SES). Ethnic background was determined by asking the country of birth of the child and for both parents. The parents were able to choose from the following six categories: “The Netherlands”, “Turkey”, “Morocco”, “Suriname”, “Netherlands Antilles” or “Other country”. The results were classified according to standard procedures established by Statistics Netherlands [35]: “Caucasian” (native-born and western migration background) and “non-Caucasian” (non-western migration background). The parent’s level of education was based on the highest level achieved and was classified as follows in accordance with international classification systems [36]: “low” (lower general secondary education, lower vocational training and primary school), “middle” (intermediate vocational training, higher general secondary training, or pre-university education), or “high” (higher vocational training or university education). If the educational level of both parents differed from each other, then the following criteria were used: If the parents’ level of education of one parent is low and the other is middle, then the education level of both parents is low; If parents’ level of education of one parent is low and the other is high, then the education level of both parents is middle; If the parents’ level of education of one parent is middle and the other is high, then the education level of both parents is middle.

#### Parental rules

Parental EBRB rules were assessed by asking the parents whether they had rules and/or agreements with their child regarding the following seven aspects: *i)* consuming snacks, *ii)* drinking SSBs, *iii)* eating fruit, *iv)* eating vegetables, *v)* eating breakfast each morning, *vi)* total hours per day spent watching television, DVDs, or other videos, and *vii)* total hours spent per day using the computer (including accessing the Internet and/or playing computer games). Parents were instructed to answer each question on a 3-point scale, with the following possible answers: “strict”, “indulgent”, or “no rules”. Parents were provided with brief definitions of these three categories: *i)* yes, and we stick to them; *ii)* yes, but we are flexible with them, and *iii)* no, we have no rules about it.

#### Energy balance-related behaviours

Parents were asked how often their child eats/consumes the following items: i) SSBs, ii) fruit, iii) vegetables, and iv) breakfast. Eight responses were possible, including “never”, “1 day a week” to “7 days a week”. In addition, parents were asked the following question: *“On days that your child drinks a SSB, how many glasses does he/she drink?”* Seven responses were possible, including “1 glass a day” to “6 glasses a day”, and “more than 6 glasses a day”.

To assess the child’s sedentary behaviour, parents were asked the following two questions: *“How many days per week does your child watch television/videos/DVDs?”* and *“How many days per week does your child use the computer (including accessing the Internet and playing computer games)?”* Eight answers were possible, including “never”, “1 day a week”, and “7 days a week”. In addition, parents were asked how much time their child spends on these activities on an average day, with the following possible answers: “less than half an hour a day”, “half an hour to 1 h per day”, “1- 2 h per day”, “2–3 h a day”, and “3 h a day or longer”.

The child’s body weight and height were determined by asking the following questions: “*What is the current weight of your child in kg (without clothing)?*” and “*What is the current height of your child in cm (without shoes)?*”.

### Data analysis

First, we calculated each child’s body mass index (BMI) based on the child’s weight and height reported by the parents. Each child’s BMI was then converted to weight status, which was classified into three categories (“not overweight”, “overweight”, or “obese”) based on international cut-off values for childhood overweight as recommended by the International Obesity Task Force [37].

Next, means and frequencies were calculated for descriptive analyses of the demographic variables, parental EBRB rules, the child’s dietary and sedentary behaviors, and the child’s BMI. The child’s age was dichotomized into either 4–7 years and 8–12 years, as we expect that the application of rules likely differs between younger (4–7 years of age) and older (8–12 years of age) primary school students. For example, children 4–7 years of age are generally more dependent on their parents and are more easily influenced by parenting behaviors, whereas children 8–12 years of age are generally better able to reflect on their own and delve more deeply into the intent behind their parents’ rules. In addition, parents of younger primary school children who are overweight or obese tend to underestimate their child’s weight status [33, 38]. EBRBs were dichotomized in accordance with Dutch standards for healthy EBRBs in children [21–23]. For food behaviors, the responses were dichotomized into “not daily consumption” and “daily consumption of fruit, vegetables, and breakfast”; the responses based on SSBs were dichotomized into “drinks an average of less than two glasses a day” and “drinks an average of two or more glasses a day”. Sedentary behavior was dichotomized into “120 min or less screen time a day on average” and “more than 120 min of screen time a day on average”.

Next, we conducted latent class analysis (LCA) using the software package Mplus 5.1 in order to empirically identify heterogeneous classes of the degree of consistency in applying parental EBRB rules across dietary and sedentary behavioral domains. LCA was used with ordered and unordered categorical variables as indicators of the latent classes. We used the seven manifest variables of applying parental rules to identify distinct typologies (i.e., profiles or clusters).

To obtain the most appropriate and most parsimonious model, we examined each of the five latent profiles by conducting a series of five nested models. This analysis was performed separately for the two age groups (i.e., 4–7 years of age and 8–12 years of age). The bootstrap likelihood ratio test (BLRT) [39] and the Bayesian information criterion (BIC) [40] are reliable and consistent statistical indicators for determining the number of classes in a LCA model [41]. BIC is a commonly used and trusted indicator for comparing models, with a lower BIC value indicating a better-fitting model. BLRT uses bootstrap samples to estimate the distribution of the log-likelihood difference test statistic. Thus, instead of assuming that the differences have a known distribution (e.g., a chi-square distribution), BLRT estimates the distribution empirically [41]. The significance of the resulting BLRT ρ value is then used to assess whether there is a significant improvement in fit between models that differ in their number of included classes. In addition, we determined the Akaike information criteria (AIC) and entropy values. Final solutions were determined by performing a careful ad hoc examination of the model selection criteria and by including substantive considerations such as class interpretability and distinctiveness, representation of reality, and scientific relevance.

Last, in this study we wanted to examine how the typologies differed with respect to covariates such as socio-demographic characteristics, the child’s EBRBs, and the child’s weight status. Thus, the LCA models with covariates had fixed class-specific item probabilities, whereas the item probabilities values were fixed at values from the LCA model without covariates. This was done to ensure that the covariate values were estimated on the basis of the established final solution of classes. To test whether socio-demographic characteristics are differentially related to the subtypes of the degree of consistency in applying parental EBRB rules, we made comparisons regarding the characteristics’ links to each class, compared with all other classes as reference class. The child’s EBRBs were divided into four categories. The responses were categorized into “less than 3 days” “3 to 4 days”, “5 to 6 days” and “7 days a week consumption of fruit, vegetables, and breakfast”; the responses based on SSBs were categorized into “none”, “1 glass”, “2 glasses” and “3 or more glasses a day”. In these analyses posterior probabilities were used as weight factors in order to account for profile membership uncertainty. Missing data (less than 10% of data) were imputed by estimate regression in SPSS.

## Results

### Study population

The socio-demographic characteristics of the children and parents, the application of parental EBRB rules, the children’s EBRBs, and the children’s weight status are summarized in Table 1. The highest consistency among parents of children 4–12 years of age was in the application of strict rules regarding eating breakfast (approximately 88%), but more than 20% of these parents reported that they have no rules regarding their child drinking SSB, more than 30% have no rules regarding using the computer and even 40% of parents reported that they have no rules regarding their child’s television watching. Parents of children 8–12 years apply less strict rules regarding consuming snacks (42.3%, p *p* < 0.001), fruit (52%, *p* < 0.001), and watching television (21.8%, *p* = 0.041) compared to parents of children 4–7 years (47.1%, 61.5%, and 24.4% respectively). Less than 45% of children 4–12 years eat vegetables daily, and about 64% is drinking 2 or more SSB daily. Finally, children 8–12 years of age more often have two or more hours screen time a day, compared to children 4–7 years of age, 40.1% and 16.5% (*p* < 0.001) respectively.

**Table 1:** Characteristics of the study population

	**Parents with a child 4–7 years of age** **N (%)**	**Parents with a child 8–12 years of age** **N (%)**	**Chi-square** ***p*-value**
**Gender of child**			
Male	1065 (51.2)	1406 (50.5)	
Female	1014 (48.8)	1380 (49.5)	0.600
**Ethnicity of the child**			
Caucasian^a^	1656 (83.8)	2286 (85.3)	
Non-Caucasian^b^	321 (16.2)	393 (14.7)	0.142
**Education level of the parents**			
Low	478 (26.9)	759 (32.0)	
Middle	763 (42.9)	990 (41.7)	
High	536 (30.2)	626 (26.4)	0.001
**Dietary behavior rules**			
Snacks			
Strict	979 (47.1)	1179 (42.3)	
Indulgent	794 (38.2)	1220 (43.8)	
No rules	306 (14.7)	387 (13.9)	< 0.001
Sugar-sweetened beverages			
Strict	844 (40.6)	1120 (40.2)	
Indulgent	647 (31.1)	982(35.2)	
No rules	588 (28.3)	684 (24.6)	0.002
Fruit			
Strict	1278 (61.5)	1449 (52.0)	
Indulgent	383 (18.4)	756(27.1)	
No rules	418 (20.1)	581 (20.9)	< 0.001
Vegetables			
Strict	1358 (65.3)	1838 (66.0)	
Indulgent	435 (20.9)	584(21.0)	
No rules	286 (13.8)	364 (13.1)	0.777
Breakfast			
Strict	1829 (88.0)	2459 (88.3)	
Indulgent	116 (5.6)	161(5.8)	
No rules	134 (6.5)	166 (6.0)	0.759
**Sedentary behavior rules**			
Watching television			
Strict	507 (24.4)	607(21.8)	
Indulgent	729 (35.1)	1064 (38.2)	
No rules	843 (40.5)	1115 (40.0)	0.041
Using the computer			
Strict	727 (35.0)	916(32.9)	
Indulgent	613 (29.5)	1003 (36.0)	
No rules	739 (35.5)	867 (31.1)	< 0.001
**Dietary behavior of the child**			
Sugar-sweetened beverages (< 2 glasses/day)	701 (35.3)	974 (36.4)	0.446
Fruit (7 days/week)	1293 (65.0)	1370 (51.0)	< 0.001
Vegetables (7 days/week)	823 (41.5)	1156 (43.1)	0.281
Eating breakfast (7 days week)	1955 (98.3)	2608 (97. 1)	0.008
**Sedentary behavior of the child**			
Total screen time (≤ 120 min per day)	1645 (83.5)	1568 (58.9)	< 0.001
**Weight status of the child**			
Not overweight	1668 (87.5)	2297 (89.4)	
Overweight	238 (12.5)	271 (10.6)	0.044

^a^Caucasian are native Dutch children (non-immigrant) or children with a western migration background

^b^Non-Caucasian are children with a non-western migration background

### Parental EBRB rules

To examine typologies regarding the degree of consistency with respect to applying parental EBRB rules across dietary and sedentary behavioral domains, on empirical grounds, the LCA models revealed that a five-class solution produced a better fit than a four-class solution, both for parents of children 4–7 years of age and for parents of children 8–12 years of age; specifically, this model yielded the lowest BIC and AIC values (see Table 2). However, due to the formation of two splinter groups in the five-class solution, this solution was highly similar to the four-class solution; therefore, we dropped one class on the grounds of parsimony, resulting in a four-class solution that was used in our subsequent analyses.

**Table 2:** Statistical indices obtained for latent profile models using one through five classes

**Number of classes in the model**	**Statistical indices**			
	**Parents of children 4–7 years of age**			
	**BIC**	**AIC**	**Entropy**	**BLRT H0 Llh-value**
**One class**	27,240.480	27,161.25	NA	NA
**Two classes**	25,052.463	24,888.913	0.80	-13,566.763***
**Three classes**	24,208.374	23,960.229	0.79	-12,415.457***
**Four classes**	23,636.762	23,304.023	0.81	-11,936.115***
**Five classes**	23,437.032	23,019.669	0.83	-11,593.011***
	**Parents of children 8–12 years of age**			
	**BIC**	**AIC**	**Entropy**	***BLRT H0 Llh-value***
**One class**	33,542.500	33,459.447	NA	NA
**Two classes**	30,797.703	30,625.664	0.76	-16,715.723***
**Three classes**	29,561.709	29,300.685	0.78	-15,283.832***
**Four classes**	29,193.882	28,843.873	0.80	-14,606.343***
**Five classes**	29,041.660	28,602.665	0.78	-14,362.936***

*BIC* Bayesian information criterion, *AIC* Akaike information criterion, *BLRT* bootstrap likelihood ratio test, *H0* null hypothesis *Llh* likelihood, *NA* not applicable

***p < 0.001

### Typologies

**Fig. 1:**
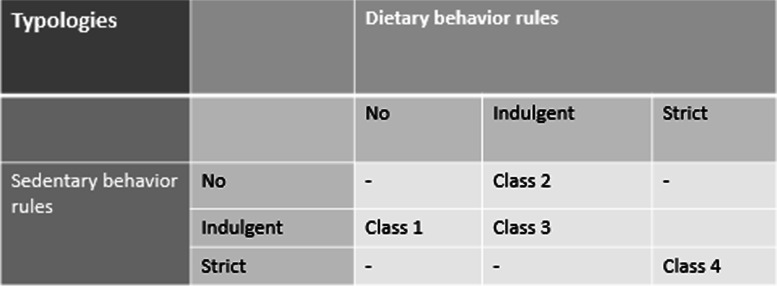
The degree of consistency in applying parental EBRB rules in the four classes

First, Fig. 1 shows the degree of consistency in applying parental EBRB rules in the four classes.

**Fig. 2:**
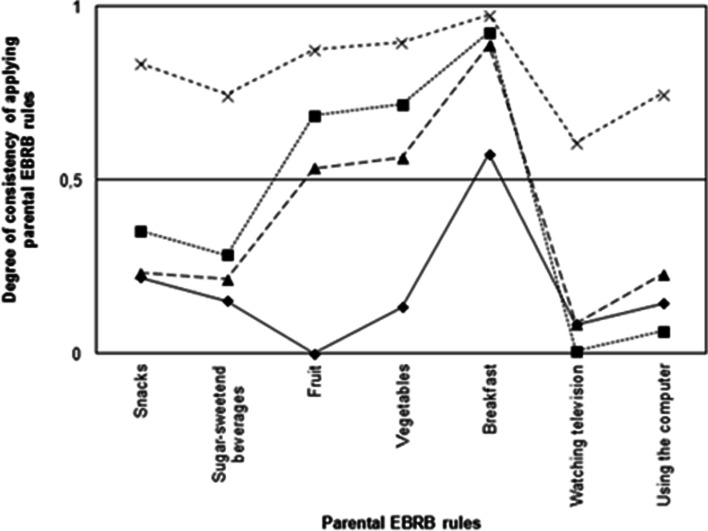
Typologies of parents of children 4–7 years applying parental EBRB rules. The degree of consistency in applying parental EBRB rules is plotted on the y-axis; 0 = no rules, 0.5 = indulgent, and 1 = strict. -◆- Class1 “no rules diet, indulgent sedentary”, *N* = 584 (27.2%); -■- class 2 “indulgent diet, no rules sedentary”, *N* = 321 (15.9%); -▲- class 3 “overall indulgent”, *N* = 708 (34.2%); -x- class 4 “overall strict”, *N* = 465 (22.6%)

**Fig. 3:**
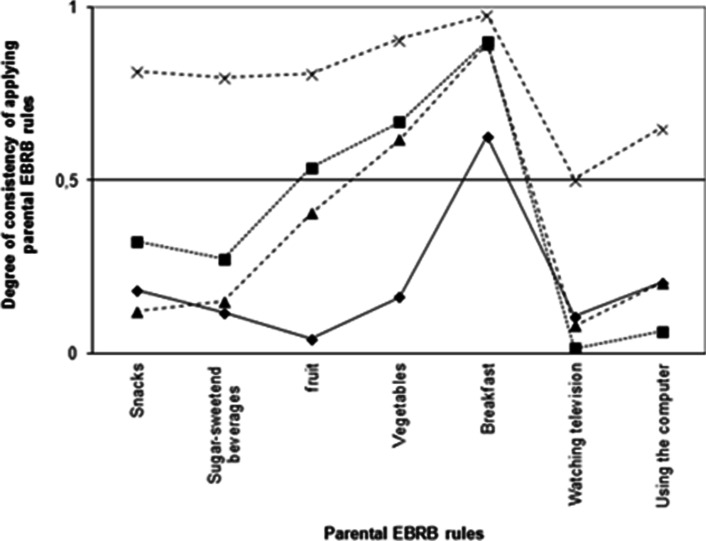
Typologies of parents of children 8–12 years applying parental EBRB rules. The degree of consistency in applying parental EBRB rules is plotted on the y-axis; 0 = no rules, 0.5 = indulgent, and 1 = strict. -◆- Class1 “no rules diet, indulgent sedentary”, *N* = 476 (17.1%); -■- class 2 “indulgent diet, no rules sedentary”, *N* = 882 (31.7%); -▲- class 3 “overall indulgent”, *N* = 1012 (36.3%); -x- class 4 “overall strict” *N* = 416 (14.9%)

Second, Figs. 2 and 3 (representing parents of children 4–7 and 8–12 years of age, respectively) show four typologies (class profiles) regarding the degree of consistency with respect to applying parental EBRB rules across dietary and sedentary behavioral domains. Both Figs. 1 and 2 show that all four classes apply indulgent or strict rules regarding eating breakfast; breakfast was therefore not taken into account when naming and interpreting the classes.

An analysis of the four typologies in both groups of parents (i.e., parents of children 4–7 years of age and parents of children 8–12 years of age revealed the overall patterns of the four classes were similar between both groups of parents, which indicates that the four classes are relatively constant between the two age groups. The distribution of children over the classes differed between the groups.

### Covariates

#### Socio-demographic covariates

Next, we tested whether the classes differed significantly with respect to the child’s age, the child’s gender, the child’s ethnicity, and/or the parents’ education level; these results are summarized in Table 3.

**Table 3:** Associations between typologies based on applying parental EBRB rules and socio-demographic covariates

			**Parents with a child 4- 7 years of age**		**Parents with a child 8- 12 years of age**	
**Covariate**	**Reference**		**β**	**SE**	**β**	**SE**
**Age of child**	Class 1	Class 2	-0.12	0.07	-0.11	0.07
		Class 3	-0.02	0.07	0.01	0.07
		Class 4	0.00	0.07	-0.15*	0.06
	Class 2	Class 3	0.10	0.07	0.12	0.06
		Class 4	0.13*	0.06	-0.03	0.05
	Class 3	Class 4	0.03	0.07	-0.15**	0.06
**Gender of child**	Class 1	Class 2	0.25	0.16	0.30	0.15
		Class 3	0.12	0.17	-0.14	0.15
		Class 4	-0.07	0.15	-0.01	0.14
	Class 2	Class 3	-0.13	0.15	-0.43**	0.13
		Class 4	-0.32*	0.13	-0.29*	0.12
	Class 3	Class 4	0.20	0.15	0.15	0.12
**Ethnicity of child**	Class 1	Class 2	-0.62**	0.24	-0.93***	0.23
		Class 3	-0.19	0.23	0.18	0.20
		Class 4	-0.12	0.20	-0.28	0.18
	Class 2	Class 3	*0.43*‡	0.22	0.75**	0.22
		Class 4	0.50*	0.20	0.65**	0.20
	Class 3	Class 4	0.08	0.22	-0.11	0.19
**Education parents**	Class 1	Class 2	-0.27**	0.08	*-0.12‡*	0.08
		Class 3	0.16‡	0.09	0.01	0.08
		Class 4	0.71*	0.11	0.17*	0.08
	Class 2	Class 3	0.11	0.08	0.12	0.07
		Class 4	0.27***	0.07	0.29**	0.06
	Class 3	Class 4	0.17*	0.12	0.16*	0.07

Class 1 = “no rules diet, indulgent sedentary”; Class 2 = “indulgent diet, no rules sedentary”; Class 3 = “overall indulgent”; Class 4 = “overall strict”; *β* beta coefficient, *SE* standard error

*p < 0.05, **p < 0.01, ***p < 0.001, and ‡*p* < 0.10. Boldface value indicate significance for both groups (4–7 years and 8–12 years), whereas values in italics indicate significance for only one group

Among parents of children 4–7 years of age, class 2 (*“indulgent diet, no rules sedentary”*) contained more parents of younger children and more parents of boys compared to class 4 (*“overall strict”*). Among parents of children 8–12 years of age, class 4 (*“overall strict”*) contained more parents of younger children compared to both class 1 (*“no rules diet, indulgent sedentary”*) and class 3 (“overall indulgent”), and class 2 (*“Indulgent diet, no rules sedentary”*) contained more parents of boys compared to both class 3 and class 4.

Similar differences were found when we examined the data based on ethnicity, the parents’ education level, and the age groups of the children (i.e., parents of children 4–7 years of age and parents of children 8–12 years of age). Class 2 (“indulgent diet, no rules sedentary”) contained more non-Caucasian parents compared to the other classes, whereas we found no effect of ethnicity in class 1, class 3, or class 4. On average, the parents in class 2 (“indulgent diet, no rules sedentary”) had the lowest level of education, followed by class 1 (“no rules diet, indulgent sedentary”), class 3 (“overall indulgent”), and class 4 (“overall strict”). In addition, the parents in class 4 (“overall strict”) had a higher average level of education than the other three classes.

#### Energy balance-related behaviors

In both groups of parents (i.e., parents of children 4–7 years of age and parents of children 8–12 years of age) we found a significant association between the degree of consistency with respect of applying EBRB rules and EBRBs among children (see Table 4). The children of parents in class 4 (“overall strict”) ate significantly more fruits and vegetables, drank fewer SSBs, and had fewer sedentary behaviour activities compared to the children of parents in the other three classes. Moreover, the children of parents in class 2 (“indulgent diet, no rules sedentary”) ate significantly more fruit and vegetables compared to the children of parents in class 1 (“no rules diet, indulgent sedentary”).

**Table 4:** Associations between typologies based on applying parental EBRB rules and prevalence of overweight among children

			**Parents with a child 4–7 years of age**		**Parents with a child 8–12 years of age**	
**Covariate**	**Reference**		**β**	**SE**	**β**	**SE**
**Drinking SSB**	Class 1	Class 2	-0.11	0.07	-0.10	0.06
		Class 3	0.09	0.07	-0.08	0.06
		Class 4	-0.34***	0.06	-0.39***	0.06
	Class 2	Class 3	0.01	0.06	*0.03*	0.05
		Class 4	-0.23**	0.06	-0.29***	0.05
	Class 3	Class 4	-0.25***	0.06	-0.32***	0.05
**Eating fruit**	Class 1	Class 2	0.24**	0.07	0.35***	0.06
		Class 3	0.11	0.07	0.25***	0.06
		Class 4	0.57**	0.10	0.70***	0.07
	Class 2	Class 3	-0.13	0.07	-0.10	0.06
		Class 4	0.33**	0.10	0.35***	0.07
	Class 3	Class 4	0.46***	0.11	0.44***	0.07
**Eating vegetables**	Class 1	Class 2	0.21**	0.08	0.18*	0.07
		Class 3	0.10	0.08	0.14*	0.07
		Class 4	0.55***	0.09	0.61***	0.09
	Class 2	Class 3	-0.11	0.07	-0.04	0.06
		Class 4	0.34***	0.08	0.43***	0.08
	Class 3	Class 4	0.45***	0.10	0.47***	0.09
**Having breakfast**	Class 1	Class 2	-0.03	0.21	0.12	0.17
		Class 3	-0.09	0.22	-0.05	0.17
		Class 4	0.24	0.24	0.42*	0.21
	Class 2	Class 3	-0.07	0.18	-0.17	0.16
		Class 4	0.26	0.21	0.30	0.20
	Class 3	Class 4	0.33	0.25	0.47*	0.21
**Sedentary behavior**	Class 1	Class 2	0.03	0.21	-0.38***	0.15
		Class 3	-0.01	0.21	-0.18	0.14
		Class 4	-0.66***	0.21	-0.94***	0.14
	Class 2	Class 3	-0.03	0.19	0.20	0.13
		Class 4	-0.69***	0.19	-0.56***	0.12
	Class 3	Class 4	-0.66**	0.21	-0.76***	0.13
**Overweight**	Class 1	Class 2	0.38	0.26	0.60*	0.26
		Class 3	0.22	0.26	0.32	0.26
		Class 4	0.14	0.25	0.27	0.25
	Class 2	Class 3	-0.16	0.21	-0.28	0.21
		Class 4	-0.24	0.20	*-0.33‡*	0.20
	Class 3	Class 4	-0.08	0.23	-0.05	0.23

Class 1 = “no rules diet, indulgent sedentary”; Class 2 = “indulgent diet, no rules sedentary”; Class 3 = “overall indulgent”; Class 4 = “overall strict”; *β* beta coefficient, *SE* standard error

*p < 005, **p < 0.01, ***p < 0.001, and ‡*p* < 0.10. Boldface value indicate significance for both groups (4–7 years and 8–12 years), whereas values in italics indicate significance for only one group

In addition, among the parents of children 8–12 years of age, the children of parents in class 2 (“indulgent diet, no rules sedentary”) adhered more closely to the limit of < 2 h screen time per day compared to the children of parents in class 1 (“no rules diet, indulgent sedentary”). Moreover, the children of parents in class 1 (“no rules diet, indulgent sedentary”) ate significantly fewer fruits and vegetables compared to the children of parents in the other three classes.

Finally, we found no effect between classes and parental rules with respect to eating breakfast.

#### Overweight

As shown in Table 4, among the parents of children 4–7 years of age we found no association between the degree of consistency with respect to applying parental EBRB rules in children and the prevalence of overweight among children. However, among the parents of children 8–12 years of age, class 2 (“indulgent diet, no rules sedentary”) contained more parents of overweight children compared to class 1 (“no rules diet, indulgent sedentary”). We also found a slight difference between class 2 (“indulgent diet, no rules sedentary”) and class 4 (“overall strict”), with class 2 containing more parents of overweight children. Finally, we found no significant difference between class 1, class 3, and class 4 with respect to parents of overweight children.

## Discussion

We identified four stable, distinct parental typologies with respect to applying dietary and sedentary behavior rules. In addition, we examined whether these typologies are associated with healthy and/or unhealthy EBRBs, and overweight among children in two non-overlapping age groups.

In addition, we found that children of parents who apply “overall strict EBRB rules” (class 4) practiced healthier behaviors (i.e., drank less SBBs, ate significantly more fruit and vegetables, and engaged in fewer sedentary activities) compared to children of parents in the other three classes. The parents in class 4 had the highest level of education. Moreover, among the children 8–12 years of age, the children whose parents apply “indulgent dietary behavior rules” (i.e., class 2 and class 3) ate more fruits and vegetables compared to children whose parents apply “no dietary behavior rules” (class 1). Therefore, we conclude that applying indulgent rules with respect to dietary behavior—although not ideal—is still better than having no rules at all.

Interestingly, children 8–12 years of age with parents in class 2 (“indulgent diet rules, no rules sedentary”) had the highest likelihood of being overweight compared to children of parents in class 1 (“no rules diet, indulgent rules sedentary’), while children 8–12 of parents in class 2 eat more fruit and vegetables, and were more likely to adhere to < 2 h of screen time per day compared to children 8–12 with parents in class 1 have. We might have expected that the children of parents in class 2 would engage in more sedentary behavior activities compared to children of parents in all of the three other classes, given that the parents in class 2 are the only parents who do not apply rules with respect to sedentary behavior activities. An explanation why children 8–12 years of parents in class 2 (“indulgent diet rules, no rules sedentary”) engaged in fewer sedentary activities compared to the children of parents in class 1 (“no dietary rules and indulgent sedentary rules”) might possibly because the parents with no sedentary rules (class 2) are either unaware or less aware of their child’s sedentary behavior activities and might therefore underestimate their child’s actual level of sedentary behavior activities. It is possibly that parents of these children also watch an excessive amount of television themselves and are therefore poor role models. This would make it difficult for such parents to apply sedentary behavior rules, particularly as the child becomes older; in addition, these parents may not be adequately aware of the importance of applying sedentary behavior rules. Class 2 contained more non-Caucasian (e.g. Turkish and Moroccan; non-western immigrants) parents and parents with the lowest level of education. This would be in line with the findings of Wijga et al., who previously reported that low maternal education, maternal overweight, and ethnicity are strongly associated with children who watch television frequently and are overweight [42]. In the Netherlands, children of parents with low SES and children of Turkish or Moroccan descent (non-Caucasian) are 2–4 times more likely to be overweight [43, 44]. It is therefore particularly important to educate these parents regarding the benefits of applying sedentary behavior rules, as these parents are an ideal target group for interventions designed to prevent and reduce overweight among children. Interventions at the policy level and group-oriented interventions are important for supporting parents. For example, ideally the same rules that parents set at home should also be applied at school, including providing healthier options. And it is important making the healthy options the easiest option to choose.

On the other hand, the degree of consistency with respect to applying EBRB rules is currently not associated with overweight in children 4–7 years of age. One possible explanation for these results is that changes in behaviors may need to occur for a longer period of time before BMI is affected. Another explanation is that the EBRB differs significantly between those two age groups. We found that compared to children 4–7 years of age, children 8–12 years of age eat less often daily fruit (65% vs. 51%), daily breakfast (98.3% vs. 97.1%) and have more than twice as often more than 2 h screentime a day (16.5% vs. 41.1%). For that reason, the difference in association with overweight seems most likely explained by the difference in EBRBs. Children 8–12 years of age have more unhealthy EBRBs compared to children 4–7 years of age.

We found that a lack of sedentary behavior rules in children 8–12 years of age—but not a lack of dietary behavior rules—is associated with overweight. This finding is consistent with the growing body of evidence suggesting that sedentary behavior (e.g., screen time) is independently and positively associated with poor health outcomes [45, 46] and is an important risk factor for childhood overweight [42, 47, 48]. Similarly, Wijga et al. also found that screen time—but none of the other behavioral factors like consumption of snacks or SSB examined was associated with childhood overweight [42]*.* Sedentary behavior has a twofold effect on energy balance. First, sedentary behavior is associated with eating more sugary, energy dense snacks and drinking more SSBs [49]; second, it reduces energy expenditure [50]. However, sedentary behavior should not necessarily be confused with a lower level of physical activity; indeed, the amount of screen time is correlated with obesity even among physically active children [51–53]. Thus, sedentary behavior rules should be applied to all children, and prevention programs must focus on educating parents with respect to the importance of establishing and consistently applying sedentary behavior rules for their children. This is consistent with the findings reported by de Jong et al. [54], who concluded that interventions should support parents in making home environmental changes, including applying sedentary behavior rules in order to reduce screen time.

### Strengths and limitations

This study’s main strengths are the large dataset used and the robust, stable classes identified. On the other hand, some limitations should be considered when interpreting our results. First, the data were obtained from questionnaires that were completed by the parents. Thus, parents who report their child’s dietary and sedentary behaviors can potentially be biased due to social desirability and/or difficulty recalling the child’s actual behavior [55, 56]. In addition, the child’s height and weight were not obtained by professionals with calibrated measuring instruments. This would have been preferred, but was practically not feasible. Thus, the calculated BMI that was potentially based upon the parents’ misperception or inaccurate home measurement of their child’s weight and height, may have resulted in an overestimation or underestimation of their child’s weight status [57–60]. Second, the response rate was 61% of nearly 16,000 parents who were invited to participate. Given that lower SES families and ethnic minorities generally have a lower rate of participation in surveys [61, 62], this might have led to a selection bias. Third, our results are based on cross-sectional data, which precludes our ability to determine causality in our results. It is therefore difficult to determine which is the cause and which is the effect, as it is likely that rules influence the EBRBs and vice versa. Fourth, the data were collected in 2009; thus, the relevance of these outcomes may have changed since then. In the past 12 years, screen time has likely increased among children, particularly given the increased availability of smartphones, tablets, and gaming consoles. A 2021 survey among parents of children 7–12 years of age found that these children use a screen more than three hours each day. Moreover, nearly half (48%) of the parents indicated that their child has been using a screen significantly more since the COVID-19 lockdown than before the lockdown, and one-fourth of responding parents (26%) reported that they now find it more difficult to limit their child's screen time. And last, on average, only 28% of the parents reported that they impose restrictions. Nevertheless, the increased screen time by children, as well as the fact that parents find it difficult to limit this time, only increases the need to establish consistent rules regarding their child’s screen time [63]. Finally, the data contained no information regarding the parents’ physical activity behavior rules. Thus, additional research and longitudinal studies are needed in order to assess the causality between typologies of parental EBRB rules, EBRBs (including physical activity), and becoming overweight. In addition, qualitative studies are needed in order to determine why some parents apply indulgent or strict EBRB rules (or lack these rules).

### Implications for practice and policy

The associations identified here provide additional evidence of the importance of the home environment, applying strict EBRB rules, and involving parents in programs designed to prevent childhood overweight. Our results may contribute to making healthcare professionals aware that children of parents who do not apply sedentary behavior rules are more likely to become overweight and underscore the need for parents to establish strict rules. Therefore, these insights may facilitate improving existing interventions designed to prevent overweight among children. In particular, parents with a low level of education and/or parents of non-Caucasian descent tend not to apply sedentary behavior rules. Therefore, effective parental education is warranted regarding the importance of applying EBRB rules—particularly sedentary behavior rules—and they must be supported with respect to applying these rules. Systematic screening of children’s health by professionals is therefore helpful, as it may provide regular opportunities to educate parents.

In addition to reducing the likelihood of childhood overweight, applying sedentary behavior rules has several other important benefits, including improving fitness, increasing self-esteem, promoting social behaviors, and improving academic performance [45].

## Conclusion

Parents’ consistency in applying dietary and sedentary behavior rules is associated with healthy dietary and sedentary behaviors, and with overweight in children. These results underscore the need for parents to establish strict rules for their children, particularly regarding sedentary behavior in order to minimize the child’s likelihood of becoming overweight. These results can serve as a starting point for developing effective strategies to prevent overweight among children.

## Data Availability

The datasets during and/or analysed during the current study available from the corresponding author on reasonable request.

## Abbreviations

SSB: Sugar-sweetened beverages
EBRB: Energy balance-related behavior
SES: Socio-economic status
BMI: Body mass index
LCA: Latent class analysis
BLRT: Bootstrap likelihood ratio test
BIC: Bayesian information criteria
AIC: Akaike information criteria
H0: Null hypothesis
Llh: Likelihood
SE: Standard error
